# rs1360780 of the *FKBP5* gene modulates the association between maternal acceptance and regional gray matter volume in the thalamus in children and adolescents

**DOI:** 10.1371/journal.pone.0221768

**Published:** 2019-08-29

**Authors:** Izumi Matsudaira, Kentaro Oba, Hikaru Takeuchi, Atsushi Sekiguchi, Hiroaki Tomita, Yasuyuki Taki, Ryuta Kawashima

**Affiliations:** 1 Department of Nuclear Medicine and Radiology, Institute of Development, Aging, and Cancer, Tohoku University, Sendai, Japan; 2 Japan Society for the Promotion of Science, Tokyo, Japan; 3 Department of Human Brain Science, Institute of Development, Aging, and Cancer, Tohoku University, Sendai, Japan; 4 Division of Developmental Cognitive Neuroscience, Institute of Development, Aging, and Cancer, Tohoku University, Sendai, Japan; 5 Department of Behavioral Medicine, National Institute of Mental Health, National Center for Neurology and Psychiatry, Tokyo, Japan; 6 Division of Medical Neuroimaging Analysis, Department of Community Medical Supports, Tohoku Medical Megabank Organization, Tohoku University, Sendai, Japan; 7 Department of Disaster Psychiatry, International Research Institute of Disaster Science, Tohoku University, Sendai, Japan; 8 Department of Psychiatry, Tohoku University Hospital, Sendai, Japan; 9 Smart-Aging Research Center, Institute of Development, Aging, and Cancer, Tohoku University, Sendai, Japan; 10 Department of Advanced Brain Science, Institute of Development, Aging, and Cancer, Tohoku University, Sendai, Japan; 11 Smart Aging International Research Center, Institute of Development, Aging, and Cancer, Tohoku University, Sendai, Japan; The University of Melbourne, AUSTRALIA

## Abstract

Investigating the effects of gene–environment interactions (G × E) with regard to brain structure may help to elucidate the putative mechanisms associated with psychiatric risk. rs1360780 (C/T) is a functional single-nucleotide polymorphism (SNP) in the gene encoding FK506–binding protein 5 (FKBP5), which is involved in the regulation of the hypothalamic–pituitary–adrenal (HPA) axis stress responses. The minor (T) allele of *FKBP5* is considered a risk allele for stress-related disorders, due to the overproduction of FKBP5, which results in impaired communication of stress signals with the HPA axis. Previous studies have reported that interactions between childhood maltreatment and the rs1360780 genotype affect structures in subcortical areas of the brain. However, it is unclear how this SNP modulates the association between non-adverse environments and brain structure. In this study, we examined the interactive effect of the rs1360780 genotype and maternal acceptance on the regional gray matter volume (rGMV) in 202 Japanese children. Maternal acceptance was assessed using a Japanese psychological questionnaire for mothers. Whole-brain multiple regression analysis using voxel-based morphometry showed a significant positive association between maternal acceptance and rGMV in the left thalamus of T-allele carriers, while a significant negative association was found in C/C homozygotes. Post-hoc analysis revealed that at or below the 70th percentiles of maternal acceptance, the T-allele carriers had a reduced thalamic rGMV compared with that of C/C homozygotes. Thus, our investigation indicated that the effect of the maternal acceptance level on brain development was different, depending on the rs1360780 genotype. Importantly, we found that the differences in brain structure between the T-allele carriers and C/C homozygotes at low to moderate levels of maternal acceptance, which is not equivalent to maltreatment. The present study contributes to the G × E research by highlighting the necessity to investigate the role of non-adverse environmental factors.

## Introduction

Gene–environment interactions (G × E) are defined as different effects of the environment on human behaviors, depending on the genotype, and vice versa. Neuroimaging studies have helped to reveal the putative mechanisms underlying the associations between candidate genetic polymorphisms and behavioral phenotypes related to the risk of psychiatric disorders [1]. However, recent studies have demonstrated that genetic polymorphisms associated with such risks are different from those associated with brain structure [2, 3]. These findings indicate that it is important to consider G × E when investigating brain structural characteristics associated with the risk of psychiatric disorders [1].

Prior G × E studies have focused on genetic polymorphisms associated with the hypothalamic–pituitary–adrenal (HPA) axis, as there is transdiagnostic evidence of the dysregulation of the HPA axis in psychiatric disorders [4]. In particular, numerous studies have demonstrated that the rs1360780 (C/T) single–nucleotide polymorphism (SNP) in the gene encoding FK506-binding protein 5 (FKBP5) modulates the association between early-life adversity and brain structure or function [5–8]. FKBP5 is a co-chaperone protein of the glucocorticoid receptor (GR) complex, which diminishes GR sensitivity to cortisol [9], and rs1360780 is a functional SNP, associated with the expression of FKBP5 [9]. The presence of the minor (T) allele results in the overexpression of FKBP5 in the context of GR activation, resulting in a prolonged cortisol stress response and impaired negative feedback of the HPA axis [9]. T/T homozygotes who experienced childhood maltreatment show reduced gray matter volumes in the insula, superior and middle temporal gyrus, hippocampus, amygdala, and anterior cingulate cortex [7]. Moreover, among major depressive disorder patients, T- allele carriers who were subjected to childhood maltreatment show reduced white matter integrity in the rolandic operculum [8]. The molecular mechanisms underlying the interaction between rs1360780 and early-life adversity are starting to be elucidated. Thus, it has been shown that early-life stress induces demethylation of a functional GR element within intron 7 of *FKBP5*, specifically in T-allele carriers [10]. This demethylation increases the FKBP5 overexpression following GR activation, resulting in further suppression of negative feedback [10]. Moreover, it has been revealed that *FKBP5* demethylation is associated with a reduced hippocampal volume [10]. Collectively, although the affected brain areas were inconsistent among various studies, neuroimaging [5–8] and epigenetic [10] evidence provides robust support for the interaction between *FKBP5* rs1360780 and early-life adversity with respect to brain structure.

However, recent G × E studies have been criticized based on associated difficulties in detecting interactions [4, 11, 12]. The effects of SNPs were small, moreover, almost all prior G × E studies focused on extreme and specific negative environments, such as childhood maltreatment [3]. Such rare combinations of genetic and environmental factors could obscure any existing interactions. In addition, it is unclear whether results from studies of childhood maltreatment are applicable to populations that lack such adverse experience during early life [13]. Therefore, it is also necessary to investigate the interaction between candidate genetic polymorphisms and non-adverse environmental factors, such as variations in parenting within the normal range [13]. Parental acceptance, which implies parental warmth, affection, love, care, nurturance, and supportiveness to a child, is an important element of positive parenting [14, 15]. There have been several studies that reported associations between positive parenting and the structure of the brain areas related to psychiatric disorders in children and adolescents [16–19]. However, it remains hitherto unknown how the interactions between the *FKBP5* rs1360780 genotype and positive parenting affect brain structures.

This study investigated how the *FKBP5* rs1360780 genotype modulated the association between positive parenting and brain structure using voxel-based morphometry. We hypothesized that subcortical areas would be affected because significant effects on the structure and function of these areas were demonstrated in previous studies of the interaction between the *FKBP5* rs1360780 genotype and early-life adversity [5–7,10]. However, there have been no prior studies investigating the interaction between positive parenting and rs1360780 with respect to brain structure, and thus, it was difficult to determine a priori the regions of interest. Therefore, we adopted a whole-brain analysis approach to test our hypothesis.

## Materials and methods

### Subjects and eligibility criteria

In contrast to prior studies, which investigated the interaction between the *FKBP5* rs1360780 genotype and childhood maltreatment in adult of European descent subjects [6, 7], all subjects of the present study were Japanese children. The reason why we chose children was that the data regarding parenting would be more accurate and the effects of various environmental factors other than parenting (e.g., lifestyle habits) on brain structure would be minimized relative to those obtained with adult samples. To our knowledge, there are no prior studies demonstrating the interaction between the *FKBP5* rs1360780 genotype and early-life adversity, such as maltreatment, in Japanese individuals. However, the robustness of interaction between the *FKBP5* rs1360780 genotype and early-life adversity has been supported by an epigenetic study [10]. Relevant epigenetic mechanisms should not differ between races, and thus, differences in the participants’ ancestry between the present study and prior studies should not crucially affect the results. We confirmed that all subjects were of Japanese ancestry by interviewing their parents.

The details regarding the initial recruitment protocol have been described previously [20]. In brief, we collected brain magnetic resonance (MR) images from 290 children (145 boys and 145 girls; age range; 5.6–18.4 years) who did not have any history of malignant tumors or head traumas involving loss of consciousness. A follow-up experiment, which included 234 children, was conducted approximately 3 years after the first experiment. The subject recruitment protocol specified that only right-handed children were eligible to participate in this project. Additionally, the Edinburgh Handedness Inventory [21] was used to confirm right-handedness. This study was conducted according to the principles of the Declaration of Helsinki (1991). Written informed consent was obtained from all participants and their parents prior to MR scanning, after the purpose and procedures used in the study were fully explained. Informed assent was also obtained from the children. Our study was approved by the Institutional Review Board of Tohoku University. Although brain MR images were obtained in both experiments, the data regarding parenting were obtained only in the first experiment, and saliva samples for genetic analysis were collected in the follow-up experiment. Therefore, to match the timing of the parenting assessment and the acquisition of the brain images, we analyzed brain images from the first experiment. Of the 234 children who participated in the follow-up experiment, 11 children were excluded because of the lack of T1-weighted images (T1WIs) in the first experiment; 15 children were excluded because of the lack of complete answers regarding maternal acceptance, and six children were excluded because of the lack of the genotype information, due to the low quality of the saliva samples. Finally, 202 participants (101 boys and 101 girls, age range: 5.7–18.4 years) were included in the present study.

During the recruitment process, children with a history of epilepsy, impaired color vision, the diagnoses of developmental and congenital disorders, routine visits to a hospital because of an illness, or routine medications (except daily drugs such as cold or allergy medications) were excluded based on the reports provided by children and their parents. To identify neurological diseases, one of the authors (YT), a professional radiologist, reviewed the T1-weighted structural images before and after processing. We also used the Child Behavior Checklist to detect behavioral problems of the subjects. Although a T-score > 70 on any subscale is considered the threshold between healthy and clinically significant cases, we did not exclude subjects based on this test score [19, 20, 22] because this criterion often excludes subjects who do not suffer from severe problems that require medication. Nevertheless, to examine whether this procedure affected the results and conclusions of the present study, we analyzed the data without subjects having a T-score > 70 on any subscale from the Child Behavior Checklist. Using this criterion, 22 additional subjects (13 C/C homozygotes and nine T-allele carriers) were excluded. The results of this analysis were mostly the same as the significant results we report in the present study, although some statistical values showed slight changes.

### Assessment of positive parenting

As an index of positive parenting, we used the answers provided by subjects’ mothers for the acceptance factor of the Family Diagnostic Test (FDT) for parents [23]. FDT is a validated Japanese questionnaire to assess the parent–child relationship and parenting style using seven factors (indifference, anxiety about parenting, disagreement with the partner, strict upbringing, hope for child’s achievement, noninterference, and acceptance). The mothers answered the questions using a Likert scale, ranging from 1 to 5 (strongly disagree to strongly agree), to describe the extent to which they agreed with each item. The original version of FDT comprises 40 question items; however, in this project, the questionnaire was shortened by randomly selecting 20 question items to reduce the burden to the mothers who were also requested to answer many other questionnaires. The acceptance factor was assessed based on five question items (including two invert items), although that in the original FDT was based on 10 question items (including five invert items). This reduction is an important limitation of the present study. However, the Cronbach’s α value of the five question items that we used was 0.72. Considering that the Cronbach’s α value of the 10 question items of the acceptance factor in the original FDT was 0.82 [23], the internal consistency of the five items we used should be reliable. Consequently, we used the total score of the five items as an index of positive parenting and referred to these data as maternal acceptance in the following sections. A higher score indicated that the mother better accepted her child.

### DNA collection and genotyping

High-molecular-weight DNA was isolated from saliva using Oragene containers (DNA Genotek, Inc., Ottawa, ON, Canada) according to the manufacturer’s protocol. The *FKBP5* polymorphism (rs1360780) was genotyped using a TaqMan assay (ID: C_8852038_10; Applied Biosystems, Foster City, CA, USA). Genotyping was conducted using a 10-μL volume containing 20 ng of genomic DNA, 5 μL of TaqMan master mix (Applied Biosystems), 0.25 μL of TaqMan assay reagents, and 2.25 μL of H_2_O. Genotyping was performed using a CFX Connect^TM^ real-time polymerase chain reaction detection system. Genotypes were scored using the algorithm and software supplied by the manufacturer (BioRad Laboratories, Hercules, CA, USA). The genotyping assays were validated in duplicate, and blanks were used as quality controls during genotyping.

In our sample, rs1360780 was in Hardy–Weinberg equilibrium (*χ*^2^ = 0.48, *p* = 0.79). We grouped the T/T homozygotes and C/T heterozygotes into one group because T is the minor allele and T/T homozygous samples were very rare among our subjects (N = 6).

### Image acquisition

All brain images were acquired using a 3-T Intera Achieva scanner (Phillips Medical Systems, Best, The Netherlands) at time point 1. T1WIs were collected using a three-dimensional, high-resolution, magnetization-prepared rapid gradient echo sequence. The imaging parameters were as follows: 240 × 240 matrix, TR = 6.5 ms, TE = 3 ms, TI = 711 ms, FOV = 24 cm, 162 slices, 1.0-mm slice thickness, and a scan duration of 483 s. Although our participants were children, sedation was not administered. Participant body motion was carefully observed by the technical staff during image aqcuisition. Scanning was repeated in the cases of excessive movement. The quality of the obtained images was evaluated by a professional radiologist (YT). Images with motion artifacts were excluded from analysis.

### Preprocessing of brain images

Preprocessing of the structural MR images was performed using the Statistical Parametric Mapping software 12 (SPM12; Wellcome Department of Cognitive Neurology, London UK) implemented in MATLAB (Math Works, Inc., Natick, MA, USA). The procedures were identical to those used in the previous study from the same project [24]. Using the segmentation algorithm implemented in SPM12, T1WIs from each subject were segmented into six tissues. In this segmentation process, default parameters were used, except that the Thorough Clean option was used to eliminate odd voxels; affine regularization was performed with the International Consortium for Brain Mapping template for East Asian brains, and the sampling distance was set at 1 mm. We then proceeded to the Diffeomorphic Anatomical Registration Through Exponentiated Lie Algebra (DARTEL) registration process implemented in SPM12. We used DARTEL-imported images of the two-tissue probability maps from the aforementioned segmentation process. First, the template for the DARTEL procedures was created using imaging data from all participants. Subsequently, the DARTEL procedures were performed for all participant images. The resulting images were spatially normalized to the Montreal Neurological Institute (MNI) space to provide images with 1.5 × 1.5 × 1.5 mm^3^ voxels. In addition, we conducted a volume change correction by modulating each voxel with the Jacobian determinants derived from spatial normalization, which allowed us to determine regional differences in the absolute amounts of brain tissue. Subsequently, all images were smoothed by convolving them with an isotropic Gaussian kernel of 8-mm full width at half maximum.

### Statistical analysis

First, we examined the independent effects of the rs1360780 genotype and maternal acceptance on brain structure using the SPM12 software. To examine the difference in regional gray matter volume (rGMV) between the rs1360780 genotypes (coded as a dummy variable, with C/C homozygotes = 0 and T-allele carriers = 1), we performed a whole-brain two-sample t-test. Further, we conducted a whole-brain multiple regression analysis to examine the association between maternal acceptance and rGMV. The subject’s age, sex, and total brain volume were entered as covariates of no interest for both analyses. Moreover, for both analyses, the initial voxel threshold was set to 0.001 uncorrected. Clusters were considered significant when they fell below a cluster-corrected p(FWE) value of 0.05.

To examine the interactive effects of the rs1360780 genotype and maternal acceptance on rGMV, a whole-brain multiple regression analysis was conducted using the SPM12 software. The rs1360780 genotype, maternal acceptance (mean-centered), and an interaction term comprising rs1360780 genotype × maternal acceptance (the product of the dummy variables for the allele and the mean-centered total value of the answers for the five question items) were entered as predictor variables. The participants’ age, sex, and total brain volume were entered as covariates of no interest. Interaction terms between predictor variables and covariates of no interest (genotype × age, genotype × sex, genotype × total brain volume, maternal acceptance × age, maternal acceptance × sex, and maternal acceptance × total brain volume) were also entered as covariates of no interest to accurately control for confounders [25]. All continuous variables were mean centered before analysis. The initial voxel threshold was set to 0.001 uncorrected. Clusters were considered significant when they fell below a cluster-corrected p(FWE) value of 0.05. For post-hoc analysis, we extracted the mean voxel value of significant clusters (the volume of the area) from each participant using the eigenvariate option in the SPM12 software. We performed the post-hoc analysis in two ways. First, simple slope analysis was used to examine the significance of the association between rGMV and maternal acceptance for each genotype. Second, we used the Johnson–Neyman method [26] to calculate the values of maternal acceptance at which rGMVs were significantly different between the genotypes. Both post-hoc tests were performed using a computational tool (http://www.quantpsy.org/interact/) developed by Preacher and colleagues [27], after the values necessary for analysis (regression coefficients and coefficients of variance of the intercept, genotype, maternal acceptance, and genotype × maternal acceptance, as well as coefficients of covariance between the intercept and genotype and between maternal acceptance and genotype × maternal acceptance) were calculated by multiple regression analysis using SPSS Statistics 24.0 (SPSS, Inc., Chicago, IL, USA).

## Results

### Participants’ characteristics

**Fig 1:**
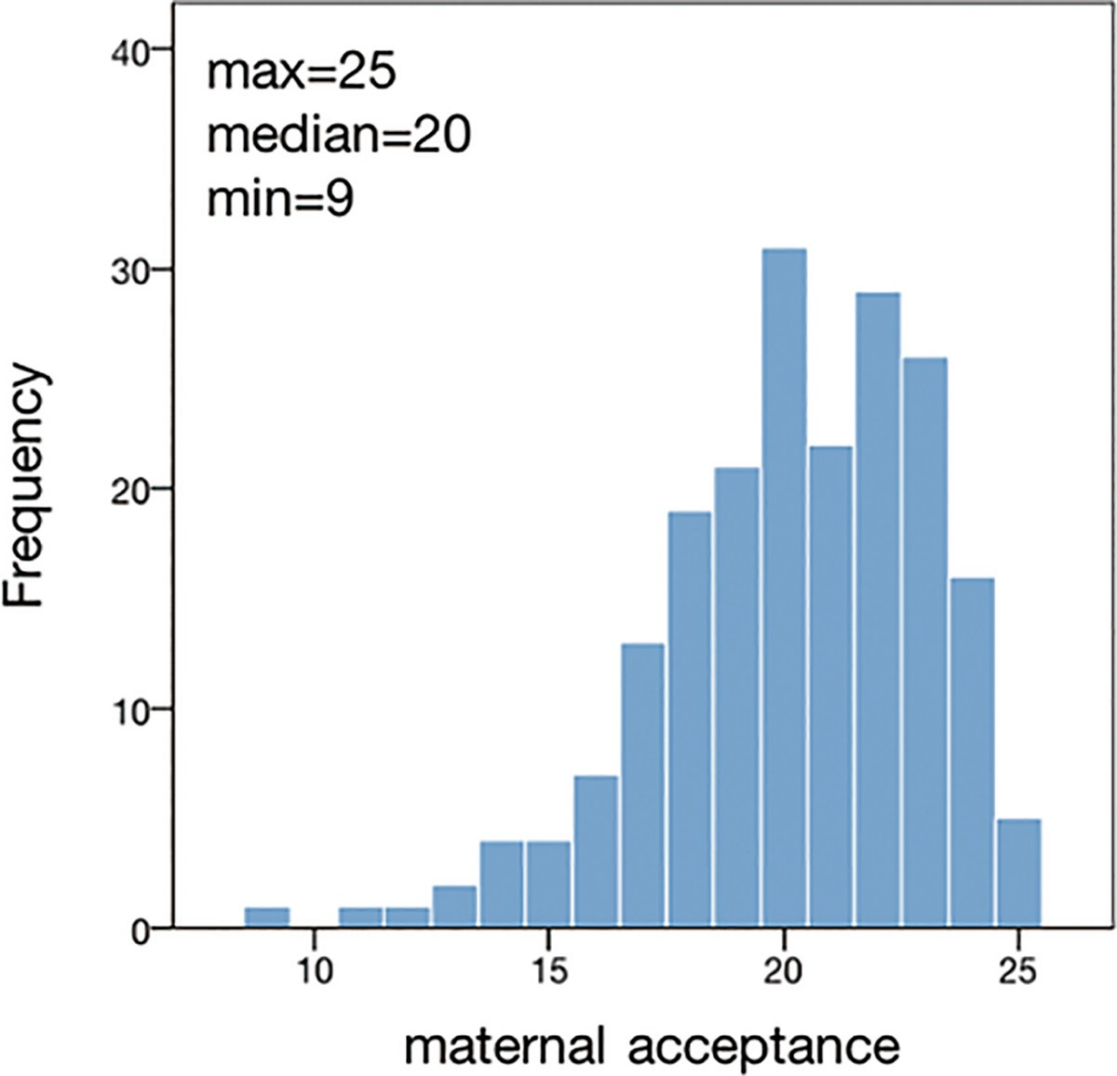
Histogram of maternal acceptance. The X-axis indicates the score of maternal acceptance, which was the total of the answers of the participants’ mothers for five question items in the acceptance factor in the Family Diagnostic Test. The Y-axis indicates the number of the participants’ mothers for each score. The maximum, median, and minimum score of maternal acceptance is also presented.

The participants’ characteristics are shown in Table 1. We found significant differences in the sex distribution between the genotypes (Table 1). We therefore entered the sex into the following analysis to correct for potential confounding effects. The distribution of maternal acceptance is shown in Fig 1.

**Table 1:** Characteristics of the subjects.

	C/C homozygotes (N = 130)		T-allele carriers (N = 72)		Analysis			Total sample	
	N	%	N	%	χ2	df	*p*	N	%
**Sex (female)**	56	43.10	45	62.50	6.99	1	0.01	101	50
	Mean	SD^a^	Mean	SD	*t*	df	*p*	Mean	SD
**Maternal acceptance**	20.26	2.87	20.01	2.91	0.58	200	0.56	20.17	2.88
**Age (years)**	11.29	3.06	11.23	2.87	0.12	200	0.91	11.27	2.99
**Total brain volume (mm**^**3**^**)**	1142.63	90.81	1116.70	20.01	1.95	200	0.05	1133.39	90.93

^a^SD, standard deviation.

### Imaging results

#### Effects of the rs1360780 genotype on rGMV

**Fig 2:**
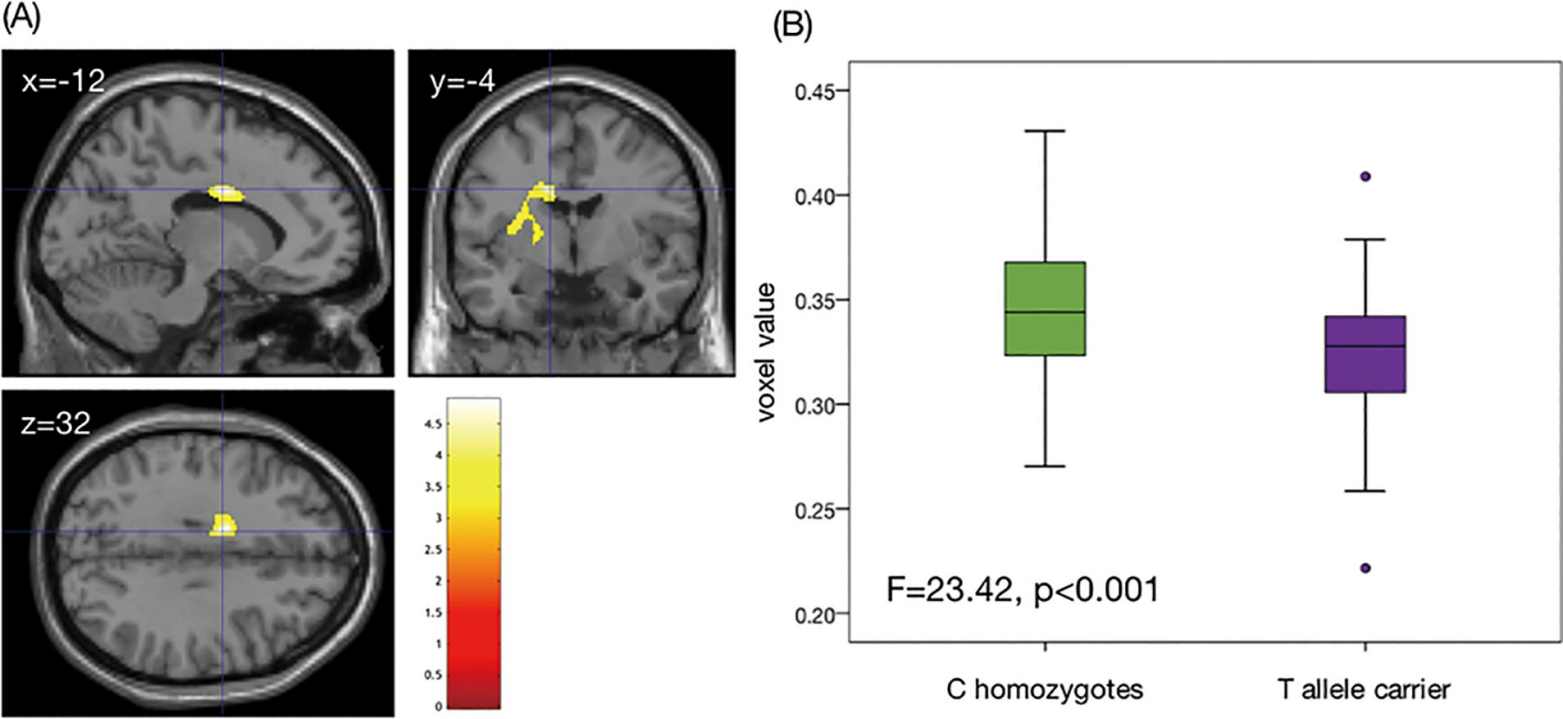
Differences in the regional gray matter volumes (rGMVs) in the middle cingulate cortex (MCC) between the rs1360780 genotypes. (A) Brain area in which the C/C homozygotes showed significantly greater rGMVs than did the T-allele carriers. The right side of the coronal image and the bottom of the axial image correspond to the right side of the brain. The color bar indicates the *T*-value. (B) Box plot indicating differences in the mean voxel value of the left MCC between the genotypes.

A two-sample *t*-test revealed significant differences in rGMVs between C/C homozygotes and T-allele carriers. As shown in Fig 2, the C/C homozygotes showed significantly greater rGMVs in the clusters, including the left middle cingulate cortex (MCC) [MNI coordinates of the peak voxel = (−12, −4, 32), *T* = 4.88, cluster size = 1,348, *p* = 0.007]. Further, there were no regions where the T-allele carriers showed significantly greater rGMVs than did the C/C homozygotes.

#### Effects of positive parenting on rGMV

**Fig 3:**
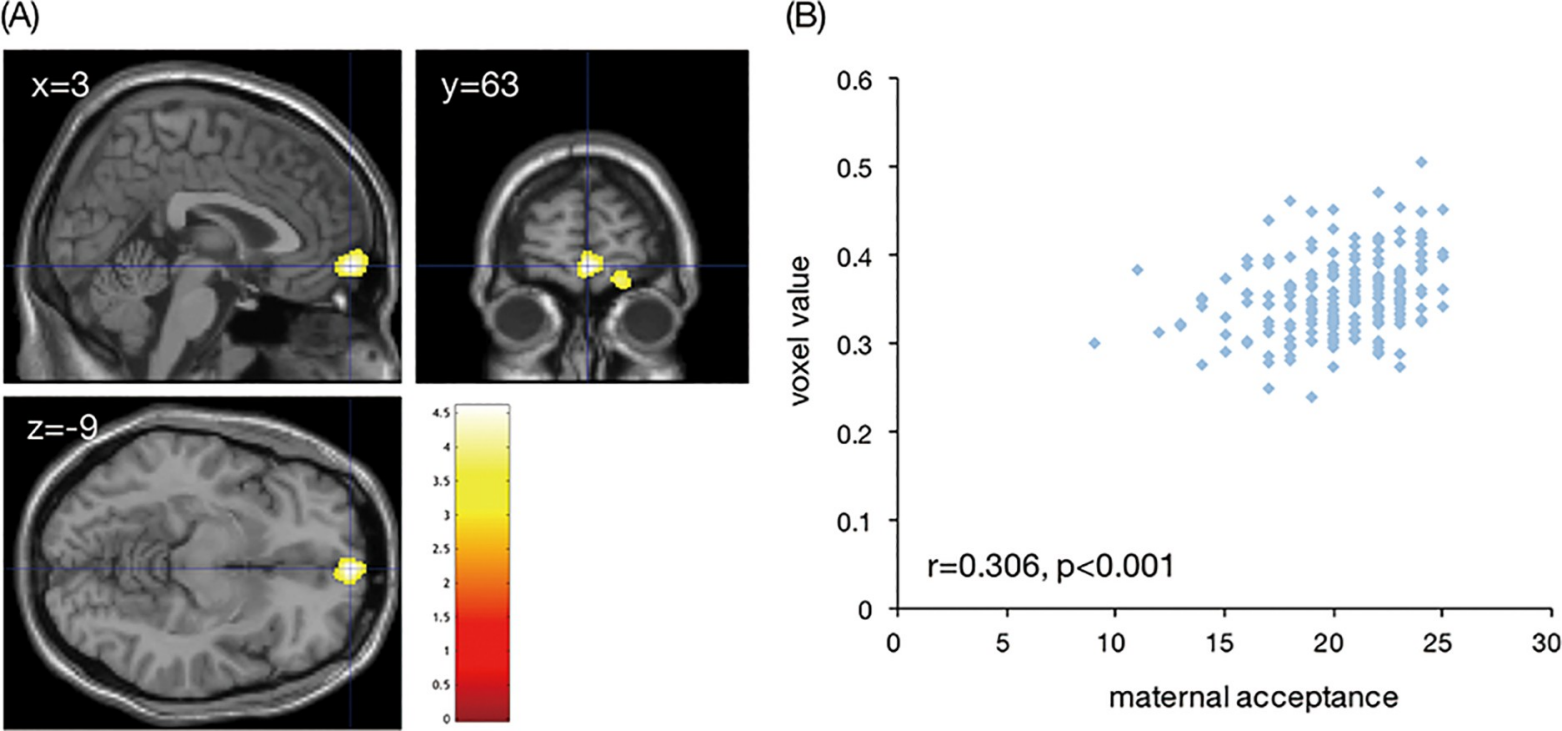
Association between maternal acceptance and the regional gray matter volume (rGMV) in the frontal pole. (A) Brain area in which the maternal acceptance and rGMV were significantly and positively correlated. The right side of the coronal image and the bottom of the axial image correspond to the right side of the brain. The color bar indicates the *T*-value. (B) Scatter plot indicating the association between the mean voxel value of the right frontal pole and maternal acceptance.

As shown in Fig 3, multiple regression analysis demonstrated a significant positive correlation between maternal acceptance and rGMV in the right frontal pole [MNI coordinates at peak voxel = (3, 63, −9), *T* = 4.59, cluster size = 932, *p* = 0.032]. However, there was no significant negative association between maternal acceptance and rGMV in any region.

### Interactive effects of the rs1360780 genotype and positive parenting on rGMV

**Fig 4:**
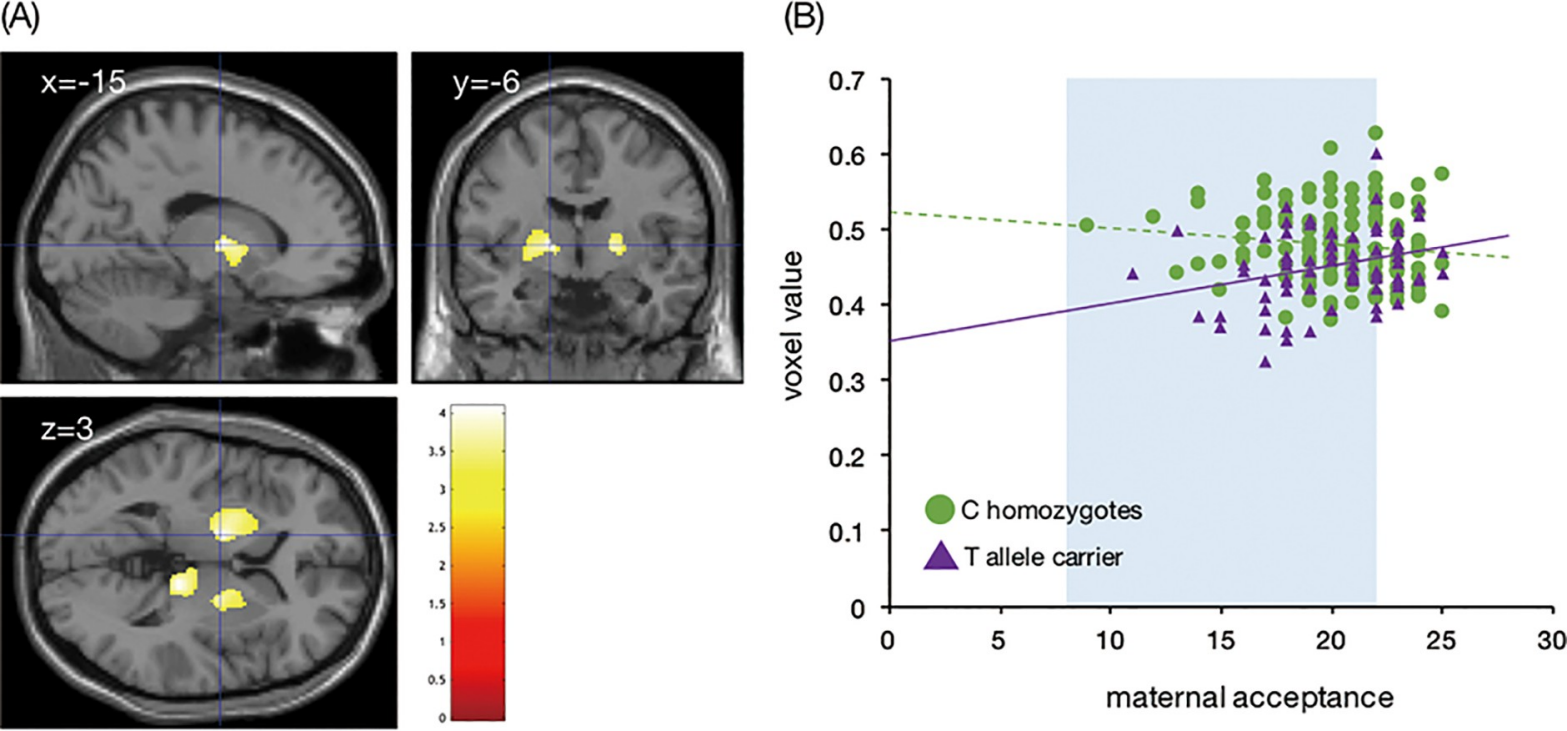
Interactive effect of the rs1360780 genotype and maternal acceptance on the regional gray matter volume (rGMV) in the left thalamus. (A) Brain area in which rGMV showed a significant interaction between the rs1360780 genotype and maternal acceptance. The right side of the coronal image and the bottom of the axial image correspond to the right side of the brain. The color bar indicates the *T*-value. (B) Scatter plot indicates that the association between maternal acceptance and rGMV in the left thalamus was modulated by the rs1360780 genotype. The violet line and violet triangles indicate T-allele carriers. The green line and green circles indicate C/C homozygotes. The highlighted area indicates the region where rGMV differences between the genotypes reached significance. Although we used mean-centered values of maternal acceptance in statistical analysis, this figure was plotted with raw values.

Multiple regression analysis using SPM12 showed a significant interactive effect of the rs1360780 genotype and maternal acceptance on rGMV in the cluster of the left thalamus (Table 2, Fig 4A). A significant interactive effect on rGMV was also observed in a similar area of the right hemisphere at a liberal statistical threshold (*p* < 0.01, uncorrected; Table 2). As shown in Fig 4B, simple slope analysis indicated a significant positive association between maternal acceptance and rGMV in the left thalamus of the T-allele carriers (simple slope = 0.005, *t* = 2.47, *p* = 0.014); in contrast, a significant negative association was observed in the C/C homozygotes (simple slope = −0.003, *t* = −1.99, *p* = 0.047). Moreover, Johnson–Neyman analysis indicated that among participants with maternal acceptance score at or below the 70th percentiles (scoring 22 or lower on the maternal acceptance scale), the T-allele carriers had significantly reduced thalamic rGMVs compared with those of the C/C homozygotes (t = 3.81, *p* < 0.001).

**Table 2:** Brain areas and Montreal Neurological Institute (MNI) coordinates that demonstrate the interaction between rs1360780 genotype and maternal acceptance.

Brain area	Peak voxel MNI coordinates			Peak level *T-* score	Cluster size (number of voxels)	Cluster *p-* value (FWE corrected)
	*x*	*y*	*z*			
**Left thalamus**	−15	−6	3	4.08	1562	0.003
**Right thalamus**	12	−28	3	3.98	712	0.078
**Right thalamus**	21	−6	2	3.72	560	0.149

The level of statistical significance was set at *p* < 0.05 and was corrected at the non-isotropic adjusted cluster level using a family-wise error (FWE) with an underlying voxel level of *p* < 0.001.

## Discussion

To the best of our knowledge, this is the first study to demonstrate the interactive effect of the *FKBP5* rs1360780 genotype and a non-adverse environmental factor on brain structure. We found a significant positive association between maternal acceptance and rGMV in the thalamus of T-allele carriers. In contrast, there was a significant negative association between rGMV in the thalamus and maternal acceptance in C/C homozygotes. Moreover, at or below the 70th percentiles of maternal acceptance, rGMVs in this area were significantly smaller in the T-allele carriers than in the C/C homozygotes.

The thalamus is a critical component of the cortical–basal ganglia–thalamic circuits, integrating various inputs related to emotions, cognition, motivation, and motor function to modulate behaviors [28]. Structural and functional abnormalities in these circuits are related to impaired cognitive control, which results in various psychopathologies, such as major depressive disorder or obsessive–compulsive disorder [29]. The regulatory role of the thalamus in the HPA axis stress response was also reported in animal studies [30, 31]. The anterodorsal thalamic nucleus in rats inhibits the adrenocorticotropic hormone release under basal, non-stressful conditions [30]. In response to chronic stress, corticosterone binds to GR in the posterior paraventricular thalamus in rats to promote feedback inhibition of the HPA axis stress response and habituation to stress [31]. Moreover, an association between glucocorticoid levels and the thalamic volume was demonstrated by imaging studies [32, 33]. It was also reported that the thalamus is one of the areas that express FKBP5 [34, 35], and the distribution of FKBP5 under basal conditions is identical to that of GR [34], which was previously detected in the thalamus using immunohistochemistry and in situ hybridization [35]. To our knowledge, there are no studies demonstrating the association between the thalamic structure in children or adolescents and maternal acceptance; however, an association between the structure and function of the thalamus and aversive parenting has been shown in several studies [36–38]. The left thalamic gray matter volume was found to correlate with childhood maltreatment in generalized anxiety disorder patients [36], and the right thalamic gray matter volume was determined to be smaller in bipolar disorder patients who experienced emotional neglect [37]. Moreover, there is an association between an increased T2 relaxation time in the thalamus and harsh corporal punishment [38]. Consequently, we believe that the interaction between the rs1360780 genotype and maternal acceptance affected rGMV in the thalamus, because this area is sensitive to emotional stimuli in the context of relationships with caregivers, among the areas that expresses GR and FKBP5.

Our results also demonstrated that rGMVs in the thalamus were smaller in the T-allele carriers than in the C/C homozygotes at almost all levels of maternal acceptance. Reduced rGMVs in T-allele subjects who experienced childhood abuse have been reported previously, although the data were based on adult samples [7]. Moreover, the rs1360780 T allele are associated with a higher cortisol reactivity because of the overproduction of FKBP5 and dysregulation of the HPA axis [9, 39, 40]. Chronic glucocorticoid production results in dysregulated expression and function of the brain-derived neurotropic factor through stimulation of GR [41, 42]. Indeed, the concentration of cortisol or its interaction with environmental factors affects brain structure during the developmental period [18, 43]. Therefore, it has been suggested that the excessive cortisol secretion in a stressful environment would influence the neural development, resulting in smaller rGMVs. Importantly, the present study demonstrated that the rs1360780 T-allele carriers showed smaller rGMVs than did the C/C homozygotes, even at a low to moderate level of maternal acceptance, which is not equivalent to extremely negative environments, such as abuse and neglect. We speculate that T-allele carriers sensitively perceive stress from relationships with caregivers because there is evidence that these individuals have an enhanced attention bias to threat [44]. Hence, even if it is not an extremely and specifically negative environment, rs1360780 T-allele carriers would regard such environment as stressful, and subsequent excessive cortisol production would affect the neural structure.

Although a prior study [7] that investigated the interaction between rs1360780 and childhood abuse showed reduced rGMVs in the insula, amygdala, temporal gyrus, anterior cingulate cortex, and hippocampus, we did not find any significant differences in these areas. These inconsistencies might be attributed to two causes. First, as mentioned previously, the environment we studied was not adverse. Early-life adversity induces *FKBP5* demethylation in intron 7 in T-allele carriers, and *FKBP5* demethylation negatively correlates with the hippocampal volume [10]. We speculate that no such epigenetic changes occurred among the T-allele carriers in our cohort. Thus, the neural outcome could differ from that occurring in the context of adversity. Second, the subjects of the study by Grabe et al. (2016) [7] were adults, whereas our cohort comprised children and adolescents. Prior studies have reported that subcortical structures, and especially the hippocampus, are influenced by early-life adversity [44, 45]. However, unlike that in adulthood, a reduction in the hippocampal volume is not observed in childhood because early-life stress prevents synaptogenesis but does not prevent synaptic pruning [46, 47]. Therefore, it is possible that the effect of the interaction between the rs1360780 genotype and maternal acceptance on the hippocampus and other structures appears after the maturation of these areas.

Interestingly, the interaction between the rs1360780 genotype and maternal acceptance found in this study was disordinal; specifically, higher maternal acceptance predicted larger thalamic rGMVs among T-allele carriers and smaller thalamic rGMVs among C/C homozygotes. There is an argument that disordinal interactions are often observed when the significance of the interaction is ostensive, particularly when sample sizes are small [12]. However, the region of significance calculated using the Johnson–Neyman method provides evidence of a true disordinal interaction [12]; therefore, we believe that the interaction we found was not a type I error. For reference, a prior study [48] demonstrated similar interaction results. Rabl et al. (2014) reported that the effect of early-life stress on hippocampal volume was modulated by genetic polymorphisms in *COMT* and *SLC6A4*. In particular, subjects with a low genetic risk (Val allele in *COMT* rs4680 and L allele in *SLC6A4* 5-HTTLPR) showed a positive association between early- life stress and the hippocampal volume, whereas those with a high genetic risk (Met allele in *COMT* rs4680 and S allele in *SLC6A4* 5-HTTLPR) showed a negative association [48]. It is challenging to explain the biological mechanism of the disordinal interaction that we detected; however, one possible explanation is as follows. Sheikh et al. [18] showed that the association between positive parenting and white matter integrity in girls was modulated by inherent cortisol levels. For example, in girls with higher cortisol levels at the age of 3, a negative correlation was found between positive parenting and fractional anisotropy in the anterior cingulate cortex at the age of 6, whereas a positive correlation was revealed in girls with lower cortisol levels [18]. Thus, Sheikh and colleagues suggested that the developing brains of children might be differentially affected by positive parenting, depending on the child’s inherent level of cortisol reactivity to stress. The results of the present study support this hypothesis. We suggest that rs1360780 C/C homozygotes and T-allele carriers would show different neural developmental processes in response to deficient maternal acceptance, such as impaired synaptic pruning in C/C homozygotes and decreased synaptogenesis in T-allele carriers. Our results indicated that when maternal acceptance was higher, C/C homozygotes and T-allele carriers showed similar thalamic rGMVs. Hence, we also speculate that there is an appropriate and desirable thalamus size and adequate positive parenting contributes to achieving this size irrespective of the rs1360780 genotype. Obviously, we cannot verify these speculations in this study. Further study is needed to investigate the neural and molecular mechanisms underlying the associations among the *FKBP5* genetic polymorphism, positive parenting, and brain structure.

We also found that T-allele carriers showed significantly reduced rGMVs in the left MCC compared with those in C/C homozygotes. This difference between the genotypes is consistent with the results of Fujii et al. [49] who showed that rGMVs in the left dorsal anterior cingulate cortex, which is part of MCC [50], were smaller in T-allele carriers than in C/C homozygotes in Japanese healthy adults. Our results indicate that the structural differences in the cingulate area between the rs1360780 genotypes previously existed during childhood.

Furthermore, we showed a significant positive correlation between maternal acceptance and rGMV in the right frontal pole (also known as Broadman area 10, or the ventromedial prefrontal cortex [51]). Several studies have demonstrated the association between parenting and the structure of this area. For example, maltreated children with post-traumatic stress disorder were found to have smaller rGMVs in the right ventromedial prefrontal cortex compared to those in maltreated children without post-traumatic stress disorder and controls [52]. Moreover, rGMV in the Broadman area10 is negatively correlated with maternal emotional warmth in heathy young adults [53]. The frontal pole has a very high gray matter growth rate during late childhood [54, 55]. Our subjects included children in the critical period of gray matter maturation, and thus, it seems plausible that higher maternal acceptance predicts an increased rGMV in the frontal pole.

Our study has several important limitations. First, the sample size was too small to examine G × E; therefore, the results of the present study should be considered preliminary. Second, we only examined the interactive effect on the intermediate phenotype, and thus, it is still unclear how differences in thalamic rGMVs between C/C homozygotes and T-allele carriers are reflected in behavioral phenotypes, such as depressive tendencies or resilient characteristics. Further studies should investigate the relationships among the rs1360780 genotype, positive parenting, brain structure, and behavior to gain a deeper understanding of individual differences in the risk of psychiatric disorders. Third, we have no data on maltreatment for this cohort. It is possible that children with lower maternal acceptance experienced aversive parenting, but we cannot verify this possibility and correct for its associated effect in the interaction analysis. In addition, we only focused on maternal acceptance as an environmental factor. There are various aspects of parenting within a normal (non-adverse) environment, such as warmth, harshness, responsiveness, and overprotection [13]. Further studies should investigate the interactions between genetic risk factors and various aspects of parenting. Finally, given the cross-sectional nature of our study, we were unable to observe the future outcomes with respect to the interaction between rs1360780 and maternal acceptance. Longitudinal studies will be necessary to elucidate the long-term effects of the interaction between genetic factors and non-adverse environments during developmental periods.

In summary, we showed that the *FKBP5* rs1360780 genotype modulated the association between maternal acceptance and rGMV in the thalamus of healthy children. To our knowledge, this is the first study to demonstrate a significant interactive effect of the rs1360780 genotype and non-adverse, positive parenting on brain structures that are associated with psychiatric disorders. The present study emphasizes the need to investigate the role of non-adverse environmental factors in imaging-based G × E research. Further studies may elucidate the mechanisms that underlie the risk of psychiatric disorders in people who do not experience early-life adversity.

## References

[1] Franke B, Stein JL, Ripke S, Anttila V, Hibar DP, van Hulzen KJE, et al Genetic influences on schizophrenia and subcortical brain volumes: large-scale proof of concept. Nat Neurosci. 2016;19: 420–431. 10.1038/nn.4228 26854805 PMC4852730

[2] Reus LM, Shen X, Gibson J, Wigmore E, Ligthart L, Adams MJ, et al Association of polygenic risk for major psychiatric illness with subcortical volumes and white matter integrity in UK Biobank. Sci Rep. 2017;7: 42140 10.1038/srep42140 28186152 PMC5301496

[3] Bogdan R, Salmeron BJ, Carey CE, Agrawal A, Calhoun VD, Garavan H, et al Imaging genetics and genomics in psychiatry: a critical review of progress and potential. Biol Psychiatry. 2017;82: 165–175. 10.1016/j.biopsych.2016.12.030 28283186 PMC5505787

[4] Bogdan R, Pagliaccio D, Baranger DA, Hariri AR. Genetic moderation of stress effects on corticolimbic circuitry. Neuropsychopharmacology. 2016;41: 275–296. 10.1038/npp.2015.216 26189450 PMC4677127

[5] White MG, Bogdan R, Fisher PM, Munoz KE, Williamson DE, Hariri AR. *FKBP5* and emotional neglect interact to predict individual differences in amygdala reactivity. Genes Brain Behav. 2012;11: 869–878. 10.1111/j.1601-183X.2012.00837.x 22979952 PMC3838302

[6] Holz NE, Buchmann AF, Boecker R, Blomeyer D, Baumeister S, Wolf I, et al Role of *FKBP5* in emotion processing: results on amygdala activity, connectivity and volume. Brain Struct Funct. 2015;220: 1355–1368. 10.1007/s00429-014-0729-5 24756342

[7] Grabe HJ, Wittfeld K, Van der Auwera S, Janowitz D, Hegenscheid K, Habes M, et al Effect of the interaction between childhood abuse and rs1360780 of the *FKBP5* gene on gray matter volume in a general population sample. Hum Brain Mapp. 2016;37: 1602–1613. 10.1002/hbm.23123 26813705 PMC6867563

[8] Tozzi L, Carballedo A, Wetterling F, McCarthy H, O'Keane V, Gill M, et al Single-nucleotide polymorphism of the *FKBP5* gene and childhood maltreatment as predictors of structural changes in brain areas involved in emotional processing in depression. Neuropsychopharmacology. 2016;41: 487–497. 10.1038/npp.2015.170 26076833 PMC5130124

[9] Binder EB. The role of FKBP5, a co-chaperone of the glucocorticoid receptor in the pathogenesis and therapy of affective and anxiety disorders. Psychoneuroendocrinology. 2009;34: S186–S195. 10.1016/j.psyneuen.2009.05.021 19560279

[10] Klengel T, Mehta D, Anacker C, Rex-Haffner M, Pruessner JC, Pariante CM, et al Allele-specific *FKBP5* DNA demethylation mediates gene–childhood trauma interactions. Nat Neurosci. 2013;16: 33–41. 10.1038/nn.3275 23201972 PMC4136922

[11] Duncan LE, Keller MC. A critical review of the first 10 years of candidate gene-by-environment interaction research in psychiatry. Am J Psychiatry. 2011;168: 1041–1049. 10.1176/appi.ajp.2011.11020191 21890791 PMC3222234

[12] Dick DM, Agrawal A, Keller MC, Adkins A, Aliev F, Monroe S, et al Candidate gene–environment interaction research: reflections and recommendations. Perspect Psychol Sci. 2015;10: 37–59. 10.1177/1745691614556682 25620996 PMC4302784

[13] Belsky J, de Haan M. Annual Research Review: Parenting and children's brain development: the end of the beginning. J Child Psychol Psychiatry. 2011;52: 409–428. 10.1111/j.1469-7610.2010.02281.x 20626527

[14] Rohner R. They love me, they love me not: a worldwide study of the effects of parental acceptance and rejection. New Heaven: HRAF Press; 2009.

[15] Seay A, Freysteinson WM, McFarlane J. Positive parenting. Nurs Forum. 2014;49: 200–208. 10.1111/nuf.12093 24898152

[16] Luby JL, Barch DM, Belden A, Gaffrey MS, Tillman R, Babb C, et al Maternal support in early childhood predicts larger hippocampal volumes at school age. Proc Natl Acad Sci U S A. 2012;109: 2854–2859. 10.1073/pnas.1118003109 22308421 PMC3286943

[17] Whittle S, Simmons JG, Dennison M, Vijayakumar N, Schwartz O, Yap MBH, et al Positive parenting predicts the development of adolescent brain structure: a longitudinal study. Dev Cogn Neurosci. 2014;8: 7–17. 10.1016/j.dcn.2013.10.006 24269113 PMC6990097

[18] Sheikh HI, Joanisse MF, Mackrell SM, Kryski KR, Smith HJ, Singh SM, et al Links between white matter microstructure and cortisol reactivity to stress in early childhood: evidence for moderation by parenting. Neuroimage Clin. 2014;6: 77–85. 10.1016/j.nicl.2014.08.013 25379418 PMC4215465

[19] Matsudaira I, Yokota S, Hashimoto T, Takeuchi H, Asano K, Asano M, et al Parental praise correlates with posterior insular cortex gray matter volume in children and adolescents. PLoS One. 2016;11: e0154220 10.1371/journal.pone.0154220 27101139 PMC4839741

[20] Taki Y, Hashizume H, Sassa Y, Takeuchi H, Asano M, Asano K, et al Breakfast staple types affect brain gray matter volume and cognitive function in healthy children. PLoS One. 2010;5: e15213 10.1371/journal.pone.0015213 21170334 PMC2999543

[21] Oldfield RC. The assessment and analysis of handedness: the Edinburgh inventory. Neuropsychologia. 1971;9: 97–113. 5146491 10.1016/0028-3932(71)90067-4

[22] Takeuchi H, Taki Y, Hashizume H, Asano K, Asano M, Sassa Y, et al The impact of parent–child interaction on brain structures: cross-sectional and longitudinal analyses. J Neurosci. 2015;35: 2233–2245. 10.1523/JNEUROSCI.0598-14.2015 25653378 PMC6705349

[23] Azuma H, Kashiwagi K, Shigeta S, Karasawa M. Family diagnostic test. Tokyo: Nihon Bunka Kagakusha; 2002.

[24] Takeuchi H, Taki Y, Asano K, Asano M, Sassa Y, Yokota S, et al Impact of frequency of internet use on development of brain structures and verbal intelligence: longitudinal analyses. Hum Brain Mapp. 2018;39: 4471–4479 10.1002/hbm.24286 29956399 PMC6866412

[25] Keller MC. Gene × environment interaction studies have not properly controlled for potential confounders: the problem and the (simple) solution. Biol Psychiatry. 2014;75: 18–24. 10.1016/j.biopsych.2013.09.006 24135711 PMC3859520

[26] Johnson PO, Neyman J. Tests of certain linear hypotheses and their applications to some educational problems. Statistical Research Memoirs. 1936;1: 57–93.

[27] Preacher KJ, Curran PJ, Bauer DJ. Computational tools for probing interactions in multiple linear regression, multilevel modeling, and latent curve analysis. J Educ Behav Stat. 2006;31: 437–448. 10.3102/10769986031004437

[28] Haber SN, Calzavara R. The cortico-basal ganglia integrative network: the role of the thalamus. Brain Res Bull. 2009;78: 69–74. 10.1016/j.brainresbull.2008.09.013 18950692 PMC4459637

[29] Peters SK, Dunlop K, Downar J. Cortico-striatal-thalamic loop circuits of the salience network: a central pathway in psychiatric disease and treatment. Front Sys Neurosci. 2016;10: 104 10.3389/fnsys.2016.00104 28082874 PMC5187454

[30] Suarez M, Perassi NI. Influence of anterodorsal thalami nuclei on ACTH release under basal and stressful conditions. Physiol Behav. 1997;62: 373–377. 10.1016/s0031-9384(97)00032-2 9251982

[31] Jaferi A, Bhatnagar S. Corticosterone can act at the posterior paraventricular thalamus to inhibit hypothalamic–pituitary–adrenal activity in animals that habituate to repeated stress. Endocrinology. 2006;147: 4917–4930. 10.1210/en.2005-1393 16809449

[32] Lau WKW, Leung MK, Law ACK, Lee TMC. Moderating effects of cortisol on neural-cognitive association in cognitively normal elderly subjects. Front Aging Neurosci. 2017;9: 163 10.3389/fnagi.2017.00163 28596732 PMC5443153

[33] Chen R, Muetzel RL, El Marroun H, Noppe G, van Rossum EF, Jaddoe VW, et al No association between hair cortisol or cortisone and brain morphology in children. Psychoneuroendocrinology. 2016;74: 101–110. 10.1016/j.psyneuen.2016.08.023 27598456

[34] Scharf SH, Liebl C, Binder EB, Schmidt MV, Muller MB. Expression and regulation of the *Fkbp5* gene in the adult mouse brain. PLoS One. 2011;6: e16883 10.1371/journal.pone.0016883 21347384 PMC3036725

[35] Morimoto M, Morita N, Ozawa H, Yokoyama K, Kawata M. Distribution of glucocorticoid receptor immunoreactivity and mRNA in the rat brain: an immunohistochemical and in situ hybridization study. Neurosci Res. 1996;26: 235–69. 9121734 10.1016/s0168-0102(96)01105-4

[36] Liao M, Yang F, Zhang Y, He Z, Song M, Jiang T, et al Childhood maltreatment is associated with larger left thalamic gray matter volume in adolescents with generalized anxiety disorder. PLoS One. 2013;8: e71898 10.1371/journal.pone.0071898 23951265 PMC3741188

[37] Duarte DG, Neves Mde C, Albuquerque MR, de Souza-Duran FL, Busatto G, Correa H. Gray matter brain volumes in childhood-maltreated patients with bipolar disorder type I: a voxel-based morphometric study. J Affect Disord. 2016;197: 74–80. 10.1016/j.jad.2016.02.068 26970268

[38] Sheu YS, Polcari A, Anderson CM, Teicher MH. Harsh corporal punishment is associated with increased T2 relaxation time in dopamine-rich regions. Neuroimage. 2010;53: 412–419. 10.1016/j.neuroimage.2010.06.043 20600981 PMC3854930

[39] Ising M, Depping AM, Siebertz A, Lucae S, Unschuld PG, Kloiber S, et al Polymorphisms in the *FKBP5* gene region modulate recovery from psychosocial stress in healthy controls. Eur J Neurosci. 2008;28: 389–398. 10.1111/j.1460-9568.2008.06332.x 18702710

[40] Luijk MP, Velders FP, Tharner A, van Ijzendoorn MH, Bakermans-Kranenburg MJ, Jaddoe VW, et al FKBP5 and resistant attachment predict cortisol reactivity in infants: gene–environment interaction. Psychoneuroendocrinology. 2010;35: 1454–61. 10.1016/j.psyneuen.2010.04.012 20547006

[41] Chen H, Lombes M, Le Menuet D. Glucocorticoid receptor represses brain-derived neurotrophic factor expression in neuron-like cells. Mol Brain. 2017;10:12 10.1186/s13041-017-0295-x 28403881 PMC5389111

[42] Numakawa T, Odaka H, Adachi N. Actions of brain-derived neurotrophic factor and glucocorticoid stress in neurogenesis. Int J Mol Sci. 2017;18 10.3390/ijms18112312 29099059 PMC5713281

[43] Dahmen B, Puetz VB, Scharke W, von Polier GG, Herpertz-Dahlmann B, Konrad K. Effects of early-life adversity on hippocampal structures and associated HPA axis functions. Dev Neurosci. 2018;40: 13–22. 10.1159/000484238 29237154

[44] Fani N, Gutman D, Tone EB, Almli L, Mercer KB, Davis J, et al *FKBP5* and attention bias for threat: associations with hippocampal function and shape. JAMA Psychiatry. 2013;70: 392–400. 10.1001/2013.jamapsychiatry.210 23407841 PMC3732315

[45] Teicher MH, Tomoda A, Andersen SL. Neurobiological consequences of early stress and childhood maltreatment: are results from human and animal studies comparable? Ann N Y Acad Sci. 2006;1071: 313–323. 10.1196/annals.1364.024 16891580

[46] Teicher MH, Samson JA, Anderson CM, Ohashi K. The effects of childhood maltreatment on brain structure, function and connectivity. Nat Rev Neurosci. 2016;17: 652–666. 10.1038/nrn.2016.111 27640984

[47] Andersen SL, Teicher MH. Delayed effects of early stress on hippocampal development. Neuropsychopharmacology. 2004;29: 1988–1993. 10.1038/sj.npp.1300528 15316569

[48] Rabl U, Meyer BM, Diers K, Bartova L, Berger A, Mandorfer D, et al Additive gene–environment effects on hippocampal structure in healthy humans. J Neurosci. 2014;34: 9917–9926. 10.1523/JNEUROSCI.3113-13.2014 25057194 PMC4107408

[49] Fujii T, Ota M, Hori H, Hattori K, Teraishi T, Sasayama D, et al Association between the common functional *FKBP5* variant (rs1360780) and brain structure in a non-clinical population. J Psychiatr Res. 2014;58: 96–101. 10.1016/j.jpsychires.2014.07.009 25088286

[50] Vogt BA. Midcingulate cortex: structure, connections, homologies, functions and diseases. J Chem Neuroanat. 2016;74: 28–46. 10.1016/j.jchemneu.2016.01.010 26993424

[51] Tsujimoto S, Genovesio A, Wise SP. Frontal pole cortex: encoding ends at the end of the endbrain. Trends Cogn Sci. 2011;15: 169–176. 10.1016/j.tics.2011.02.001 21388858

[52] Morey RA, Haswell CC, Hooper SR, De Bellis MD. Amygdala, hippocampus, and ventral medial prefrontal cortex volumes differ in maltreated youth with and without chronic posttraumatic stress disorder. Neuropsychopharmacology. 2016;41: 791–801. 10.1038/npp.2015.205 26171720 PMC4707825

[53] Yang J, Wei D, Wang K, Yi Z, Qiu J. Regional gray matter volume mediates the relationship between maternal emotional warmth and gratitude. Neuropsychologia. 2018;109: 165–172. 10.1016/j.neuropsychologia.2017.12.017 29241650

[54] Sowell ER, Peterson BS, Thompson PM, Welcome SE, Henkenius AL, Toga AW. Mapping cortical change across the human life span. Nat Neurosci. 2003;6:309–315. 10.1038/nn1008 12548289

[55] Shaw P, Kabani NJ, Lerch JP, Eckstrand K, Lenroot R, Gogtay N, et al Neurodevelopmental trajectories of the human cerebral cortex. J Neurosci. 2008;28: 3586–3594. 10.1523/JNEUROSCI.5309-07.2008 18385317 PMC6671079

